# Comparing Conduction System Pacing to Biventricular Upgrade in Pacemaker-Induced Cardiomyopathy: A Retrospective Observational Study

**DOI:** 10.3390/jcm14217745

**Published:** 2025-10-31

**Authors:** Bernadett Miriam Dobai, Balázs Polgár, Márk Gémesi, Manuella Bogdan, Nikolett Vigh, Mirjam Turáni, Gábor Zoltán Duray, Péter Bógyi

**Affiliations:** 1 Doctoral School of Medicine and Pharmacy, George Emil Palade University of Medicine, Pharmacy, Science and Technology of Targu Mures, 540139 Targu Mures, Romania; 2 Centre for Translational Medicine, Semmelweis University, 1085 Budapest, Hungary; 3 Central Hospital of Northern Pest—Military Hospital, 1134 Budapest, Hungary; 4 Doctoral College, Semmelweis University, 1085 Budapest, Hungary; 5 Heart and Vascular Center, Semmelweis University, 1124 Budapest, Hungary

**Keywords:** pacemaker-induced cardiomyopathy, conduction system pacing, his-bundle pacing, left bundle branch area pacing, biventricular pacing, cardiac resynchronization therapy, heart failure, upgrade procedure

## Abstract

**Background/Objectives**: Pacemaker-induced cardiomyopathy (PICM) develops in up to 30% of patients with chronic right ventricular pacing. While biventricular (BIV) upgrade is the conventional strategy, conduction system pacing (CSP) offers a physiologic alternative recently endorsed by the 2025 ESC/EHRA Consensus Statement. However, comparative evidence in PICM is limited. Therefore, we aimed to compare outcomes of PICM patients undergoing CSP versus BIV upgrade. **Methods**: This retrospective analysis included consecutive PICM patients who were upgraded to CSP or BIV between 2022 and 2024 at a single, experienced center. Follow-up averaged >19 months. Clinical outcomes, lead performance, echocardiographic parameters, complications, and quality of life (QoL) were evaluated. **Results**: Sixty-three patients were included (CSP: 26; BIV: 37). Mean age and sex distribution were similar; both groups had wide paced QRS complexes and a high ventricular pacing burden. Baseline left ventricular ejection fraction (LVEF) was lower in BIV patients (29 ± 7% vs. 35 ± 6%, *p* = 0.01). Procedure duration was comparable, but fluoroscopy was shorter with CSP. QRS duration narrowed significantly in both groups (CSP: 163 ± 28→132 ± 12 ms; BIV: 171 ± 23→140 ± 18 ms; both *p* < 0.05). During follow-up, LVEF improved (CSP: 41 ± 8%; *p* = 0.008; BIV: 39 ± 8%, *p* = 0.0001), as did NYHA class, with no significant intergroup differences. The rates of heart failure hospitalization, all-cause mortality, and QoL were similar. Notably, 34.6% of CSP patients retained their existing generator, suggesting procedural and economic benefits. **Conclusions**: CSP is a feasible and potentially cost-efficient alternative to BIV upgrade in PICM, with comparable improvements in ventricular function, symptoms, and clinical outcomes. Larger prospective trials are warranted.

## 1. Introduction

Nearly one million people worldwide develop cardiac conduction system disease each year and require permanent pacing to prevent life-threatening or symptomatic bradycardia [1]. Although right ventricular (RV) pacing is an effective therapy for bradycardia, up to 30% of patients may subsequently develop pacemaker-induced cardiomyopathy (PICM)—a condition driven by electrical dyssynchrony and progressive adverse ventricular remodeling, leading to left ventricular (LV) dysfunction, increased heart failure hospitalizations, and reduced survival [2].

Cardiac resynchronization therapy or biventricular (BIV) pacing was validated as an upgrade strategy for patients with RV pacemakers and LV dysfunction, with multicenter studies demonstrating echocardiographic and clinical improvement [3,4,5,6,7]. However, BIV pacing requires placement of a coronary sinus lead, which can be technically challenging, prolong fluoroscopy exposure, and is not always feasible due to unfavorable venous anatomy or lead instability [8,9].

Conduction system pacing (CSP), including His-bundle pacing (HBP) and left bundle branch area pacing (LBBAP), has emerged as a physiological alternative, producing a ventricular activation pattern similar to normal conduction [10]. CSP has been endorsed by the 2025 ESC/EHRA consensus statement as an upgrade option in selected patients, based primarily on evidence from single-arm observational studies showing favorable electrical, structural, and clinical outcomes [11].

Despite growing adoption, direct head-to-head comparisons of CSP and BIV in the PICM upgrade setting are scarce. To date, only two single-center studies have directly compared the two strategies, reporting greater improvements in LV function in CSP patients and similar symptomatic responses in both groups [12,13].

The present study contributes to the limited comparative evidence on CSP and BIV upgrades in PICM, representing the third cohort to date that addresses this question. Beyond procedural and echocardiographic outcomes, it uniquely integrates functional, clinical, and quality-of-life assessments, as well as cost analysis between the two strategies.

## 2. Materials and Methods

### 2.1. Study Population

This study retrospectively analyzed consecutive patients diagnosed with pacemaker-induced cardiomyopathy (PICM) from October 2022 to October 2024 who underwent cardiac implantable electronic device (CIED) upgrade to either biventricular (BIV) or conduction system pacing (CSP). PICM was defined as a decline in left ventricular ejection fraction (LVEF) of ≥10 percentage points from a baseline of ≥50%, resulting in a follow-up LVEF of <50% in patients with high right ventricular pacing (VP) burden ≥ 40% in the absence of other identifiable causes of cardiomyopathy [2].

### 2.2. Procedure Description

The choice between conduction system pacing and biventricular upgrade was made by the implanting physicians in accordance with the EHRA Consensus Statement [14], taking into account factors such as native QRS duration, existing lead configuration, remaining generator longevity, the need for defibrillator therapy, and patient preferences through a shared decision-making process.

Conduction system pacing upgrade procedures were performed using either Medtronic Select Secure 3830 lumenless (Medtronic plc, Minneapolis, MN, USA), active-fixation helix leads or Biotronik Solia S60 stylet-driven leads (SDL, Biotronik SE & Co. KG, Berlin, Germany). The LBBAP implantation was routinely carried out following the standard CSP implantation technique previously described in the literature [14,15,16,17,18,19]. First, the tricuspid annulus was localized by contrast injection. Next, the sheath was positioned just distal to the tricuspid valve, and pace mapping was performed at the right side of the interventricular septum. A favorable-paced QRS morphology at the penetration site was defined as a QS complex with a notched nadir in V1 resembling a “W pattern.” Discordant QRS polarity in leads II and III was accepted for fixation, as well as QS complexes or R waves in these leads. At this site, the lead was advanced across the RV septum by rapid rotations. During the screw-in process, fluoroscopy, paced QRS morphology, fixation beats, pacing threshold, and lead impedance were continuously monitored to determine optimal lead depth. After deployment, an output-dependent QRS transition was observed to confirm capture of the conduction system. The achieved pacing type was classified according to the latest EHRA consensus statement [14]. In the event of a failed LBBAP, HBP was attempted.

The biventricular pacing upgrade procedure was performed using a standard technique. Left ventricular leads were implanted via the coronary sinus, with preference for positioning in the basal lateral or posterolateral veins. Quadripolar LV leads were employed in the majority of patients (94.6%, 35 of 37) to optimize pacing configurations and minimize the risk of phrenic nerve stimulation.

### 2.3. Data Collection and Follow-Up

All patients underwent standardized clinical and echocardiographic assessments at baseline and during regular follow-up. At the 6-month follow-up, the quality of life was assessed with the EQ-5D-5L questionnaire which evaluates mobility, self-care, usual activities, pain or discomfort, and anxiety or depression. Each domain is rated on five levels reflecting the severity of problems in that area. In addition, patients rated their overall health status on the visual analogue scale ranging from 0 to 100 [20,21]. The assessed outcomes included changes in left ventricular ejection fraction (LVEF), left ventricular end diastolic diameter (LVEDD), New York Heart Association (NYHA) functional class, heart failure hospitalizations, all-cause mortality, and device-related complications. Procedural, lead parameters, and pacing burden were also recorded. Procedure costs, including device-related expenses (generator, sheath, and leads) and procedural material costs, were obtained from hospital billing records and compared per patient between CSP and BIV upgrades.

### 2.4. Statistical Analysis

Continuous variables were expressed as mean ± standard deviation or as median with interquartile range. Categorical variables were summarized as counts and percentages. We assessed normality by the Shapiro–Wilk test and compared continuous data using independent *t*-tests or Mann–Whitney U tests (and paired *t*-tests or Wilcoxon signed-rank tests for within-group analyses). Categorical and ordinal variables were analyzed by chi-square or Fisher’s exact tests (with Kruskal–Wallis tests for overall ordinal comparisons and 2 × 2 Fisher’s exact tests for class-by-class transitions).

Patients were followed up regularly until the occurrence of the HF hospitalization, or were censored at the time of last follow-up or death.

Time-to-event outcomes were evaluated via Kaplan–Meier survival curves, and multivariable Cox regression was applied to adjust for baseline LVEF imbalance.

Absolute change in LVEF (ΔLVEF) was analyzed using analysis of covariance (ANCOVA), with treatment group as the independent variable and baseline LVEF as a covariate. Model assumptions were confirmed by visual inspection. A two-tailed *p* < 0.05 defined statistical significance. Given the modest sample size and event rate, propensity score matching was not performed to avoid loss of power and overfitting.

All statistical analyses were conducted in R version 4.5.0.

## 3. Results

### 3.1. Baseline Characteristics

Between January 2022 and October 2024, a total of 63 patients with pacing-induced cardiomyopathy underwent device upgrade to either biventricular pacing (*n* = 37) or conduction system pacing (*n* = 26).

Both groups were similar with respect to age (75.9 ± 6.3 vs. 75.0 ± 7.4 years), sex distribution (72.0% vs. 86.5% male), primary implant indication, ventricular pacing burden, comorbidities, baseline QRS duration, and guideline-directed heart failure therapies. Echocardiography revealed a higher baseline LVEF in the CSP group (CSP: 34.5 ± 8.0% vs. BIV: 29.7 ± 7.6%, *p* = 0.01), with no significant differences in chamber dimensions, right ventricular function, or tricuspid regurgitation grade (Table 1).

### 3.2. Procedural and Clinical Outcomes

Procedural time was similar between groups, while fluoroscopy was shorter in CSP (7.1 ± 7.3 vs. 8.6 ± 5.1 min, *p* = 0.03). Two BIV procedures were converted to CSP due to failure to obtain an adequate coronary sinus lead position (final cohort: 26 CSP, 37 BIV; Table 2). LBBAP was achieved in 92% of CSP cases, with a mean V6 R-wave peak time of 82.5 ± 15.9 ms (Table 3).

Complications were rare: one intraoperative ventricular tachycardia in the CSP arm and one coronary sinus dissection in the BIV group occurred, both without further consequences. During follow-up, one CSP patient required lead revision due to elevated thresholds of the LBBAP lead; no other procedural or device-related complications occurred.

**Figure 1:**
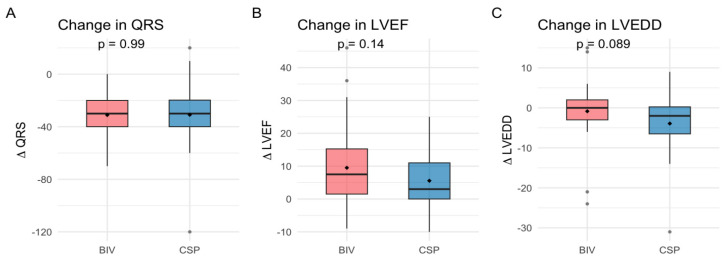
Changes in QRS duration (**A**), left ventricular ejection fraction (LVEF) (**B**), and left ventricular end-diastolic diameter (LVEDD) (**C**) after upgrade to biventricular pacing (BIV) or conduction system pacing (CSP).

**Figure 2:**
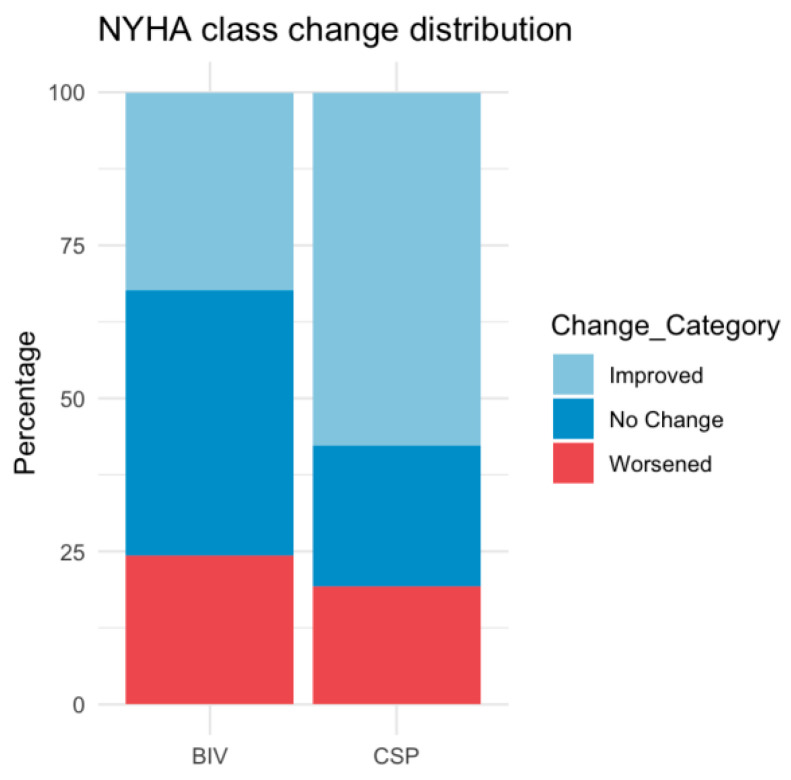
Distribution of New York Heart Association (NYHA) class change after upgrade to biventricular pacing (BIV) or conduction system pacing (CSP).

QRS narrowing, LVEF, NYHA class improvement, and LVEDD reduction were comparable between upgrade procedures (Table 2 and Table 3, Figure 1 and Figure 2), with both groups showing significant improvement from baseline to follow-up (LVEF: CSP *p* = 0.008, BIV *p* = 0.005; NYHA CSP *p* = 0.01, BIV *p* = 0.005, LVEDD: CSP *p* = 0.02; BIV *p* = 0.03). After ANCOVA adjusting for baseline LVEF, the adjusted mean improvement (ΔLVEF) was 6.6% (95% CI 2.2–11.0) in the CSP group and 8.8% (95% CI 5.2–12.4) in the BIV group. The adjusted between-group difference was −2.2% (95% CI −7.0 to 2.6; *p* = 0.38), indicating no significant difference in LVEF improvement. Additionally, baseline LVEF was associated with ΔLVEF (β = −0.375, *p* = 0.03), indicating that patients with lower baseline EF experienced greater improvement Responder rates (≥10% LVEF increase) did not differ significantly (36.1% BIV vs. 28.0% CSP, *p* = 0.58).

### 3.3. Heart Failure Hospitalization and All-Cause Mortality

**Figure 3:**
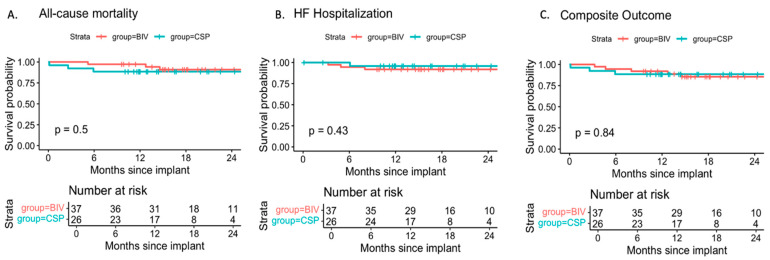
Freedom from (**A**) All-cause mortality, (**B**) heart failure (HF) hospitalization, and (**C**) the composite of death or HF hospitalization in patients with CSP or BIV upgrade. Survival probabilities are plotted over a 24-month period since device upgrade. Risk tables below each panel indicate the number of patients at risk at 6-month intervals. Differences between groups were compared using the log-rank test. No statistically significant differences were observed.

During a mean follow-up of 19.5 ± 8.1 months, the rates of all-cause mortality, heart failure hospitalization, and the composite of both events were similar between CSP and BIV upgrades. Adjustment for baseline LVEF, which was lower in the BIV group, did not alter these findings (Figure 3, Table 4). In this model, each 1% increase in baseline LVEF was associated with a 9% relative reduction in the risk of heart failure hospitalization (HR 0.91 per %, *p* = 0.18).

### 3.4. Quality of Life

Quality-of-life scores and domain responses at 6-month follow-up were similar between the CSP and BIV groups, with no significant differences observed in any measure between the groups (Table 5).

### 3.5. Financial Cost–Benefit

In our cohort, the mean procedural cost of upgrade systems was 41% lower in CSP compared to BIV upgrades. This difference was explained mainly by device selection: the existing generator could be retained in 34.6% of CSP patients due to sufficient remaining longevity, and ICD upgrade was avoided in 76.9%.

## 4. Discussion

### 4.1. Summary of Main Findings

In this retrospective, single-center observational study, we compared conduction system pacing (CSP) with biventricular pacing (BIV) as upgrade strategies for patients with pacemaker-induced cardiomyopathy (PICM). The principal findings are that: (1) both strategies achieved significant improvements in left ventricular ejection fraction (LVEF), ventricular dimensions, and New York Heart Association (NYHA) functional class; (2) quality of life and clinical outcomes, including heart failure hospitalization (HFH) and all-cause mortality (ACM), were similar between groups; and (3) retaining of the existing generator was feasible in 34.6% of CSP patients—a novel observation not previously reported in this population.

### 4.2. Comparison with Prior Studies

Biventricular pacing has been the standard upgrade strategy for patients with PICM. Multicenter observational studies have consistently demonstrated symptomatic and echocardiographic improvements with BIV, and the randomized Budapest-CRT Upgrade trial provided strong evidence, showing significant reductions in HFH and ACM in addition to reverse remodeling [3,4,5,22]. The benefit of BIV upgrade is similar in patients with atrial fibrillation [23]. However, the approach can be limited by the technical challenges of coronary sinus lead placement, as anatomical variations may lead to suboptimal or impossible lead positioning [8,9].

More recently, CSP via HBP or LBBAP has emerged as a physiologic alternative for BIV [24,25]. Multiple single-arm studies and meta-analyses suggest that CSP achieves similar or even superior resynchronization and remodeling compared with BIV, while maintaining comparable rates of HFH and ACM [26]. Only two prior head-to-head studies directly compared CSP and BIV upgrades in PICM, both reporting greater improvements in LVEF and LVEDD with CSP, with no difference in HFH or ACM [12,13]. In our larger population with follow-up beyond 19 months, there was no difference in clinical outcomes, and echocardiographic improvements were similar between groups. This similarity was likely influenced by the higher baseline LVEF in the CSP arm, which was independently associated with smaller gains, whereas lower baseline LVEF predicted greater remodeling.

Responder rates (≥10% LVEF improvement) were modest—36.1% in the BIV group and 28.0% in the CSP group—lower than the 60% typically reported in de novo CRT trials [27,28]. This likely reflects the higher baseline LVEF in the CSP group, along with the greater comorbidity burden and the high prevalence of ischemic cardiomyopathy in our cohort, all of which are known to attenuate reverse remodeling [29]. Notably, both upgrade strategies demonstrated safety, as no major late complications were observed, lead thresholds remained stable, and our findings align with those of a recent meta-analysis, which reported low complication rates for CRT (3.3%) and CSP upgrades (1.8%) [30].

Quality of life has not been systematically evaluated in previous studies of PICM upgrades. In our cohort, both CSP and BIV upgrades resulted in comparable patient-reported quality of life, consistent with the parallel improvements observed in LVEF and NYHA class. However, overall QoL remained suboptimal, with low self-reported health scores (46.5 ± 34.9 for CSP vs. 41.6 ± 31.9 for BIV; *p* = 0.46) and frequent functional limitations in both groups (42–54%, *p* > 0.05). Although only 10 patients were implanted in 2022, when COVID-19-related restrictions and psychological stress may have affected perceived well-being, this factor should be considered when interpreting the QoL score. Despite these potential confounders, the similar QoL outcomes between upgrade strategies suggest that CSP offers comparable patient-centered benefits to conventional BIV. At the same time, residual symptoms likely reflect the underlying heart failure burden and comorbidities, as previously reported in device-treated populations [31,32,33,34].

Absence of appropriate ICD therapy in our study is in line with the very low rate of malignant ventricular arrhythmias in CRT upgrade populations [3,4]. Observational and randomized data suggest that routine defibrillator implantation may not be necessary in non-ischemic cardiomyopathy patients without additional arrhythmic risk factors [35,36].

### 4.3. Mechanistic Considerations

The comparable remodeling and functional recovery between the CSP, particularly the LBBAP and BIV groups, may be explained by both approaches’ ability to restore ventricular synchrony [6]. CSP directly recruits the His–Purkinje system, producing LV activation patterns similar to those of intrinsic conduction, whereas BIV achieves resynchronization through the fusion of LV and RV pacing, as demonstrated in studies comparing LV activation patterns in CRT and CSP [10]. Clinically, these electrical advantages have been linked to larger LVEF gains with CSP versus BIV in some upgrade PICM cohorts, although most data are from single-arm observational studies.

In PICM, where dyssynchrony is pacing-induced, either strategy could reverse the underlying pathophysiology, though comorbidities and myocardial substrate may modulate the degree of benefit. Long-term follow-up is required to determine whether the similar short- to mid-term outcomes observed here persist and whether any differences emerge in the durability of response, HFH risk, or survival [37].

### 4.4. Cost Implications

A novel finding of our study is that existing generators can be reused in over one-third of CSP upgrades, whereas all BIV patients require new CRT devices. This selective reuse, together with the fact that ICD upgrades were avoided in more than three-quarters, highlights a potential cost advantage of CSP. Although specialized delivery sheaths add expense, the overall balance suggests greater cost-efficiency compared with BIV upgrades.

Another practical consideration concerns the potential presence of abandoned leads after generator reuse. Although concerns may arise regarding MRI compatibility and potential complications during future transvenous lead extractions, recent evidence suggests that MRI examinations can be performed safely when appropriate institutional protocols are followed [38,39,40].

Moreover, lead extraction is typically undertaken in younger patients, with an average reported age of around 65 years, whereas the mean age in our CSP upgrade cohort was 76 years [41]. Consequently, the clinical relevance of these concerns is limited in this elderly population, and from a cost-efficiency perspective, they do not justify the implantation of a larger and more expensive CRT generator solely to avoid a retained lead.

### 4.5. Clinical Implications

Our findings support CSP as a physiologic alternative to BIV for PICM upgrades, offering comparable functional and clinical outcomes with added procedural efficiency and potential cost savings. CSP may be particularly beneficial in patients with unfavorable coronary sinus anatomy, venous occlusion, or when generator reuse is feasible. Until randomized data are available, device selection should be individualized, taking into account patient preferences, comorbidities, and operator expertise.

## 5. Limitations

As a retrospective, single-center analysis, the present study may have limited generalizability to other populations or centers with different clinical practices. The selection of upgrade strategy (CSP or BIV) was not randomized but guided by clinical judgment, introducing a potential risk of selection bias despite statistical adjustment for confounding variables, including baseline LVEF.

A standardized definition of PICM is lacking; the current study focused on patients with a persevered baseline systolic function who developed pacing-induced LV dysfunction, which may limit comparability with studies using alternative definitions.

Coronary venous anatomy was not assessed prior to CSP implantation and therefore did not influence group allocation, although two crossover cases from CRT to CSP occurred due to unfavorable sinus coronarius anatomy.

Due to the small sample size and the low number of clinical events, this study was underpowered for hard clinical outcomes. Therefore, only large between-group differences could have been detected with sufficient statistical power, and the findings related to HFH and ACM should be interpreted with caution.

Baseline QoL EQ-5D-5L assessment was not available; therefore, only intergroup comparisons at follow-up could be performed.

Larger, multicenter, randomized studies are warranted to confirm these findings and further refine patient selection criteria for CSP versus BIV upgrades.

## 6. Conclusions

In patients with PICM, CSP upgrade represents a feasible and potentially cost-efficient alternative to BIV. CSP demonstrated favorable procedural characteristics and was associated with comparable improvements in left ventricular function, symptomatic status, and major clinical outcomes, including HFH and ACM. These findings may support its consideration as an upgrade strategy, although confirmation in adequately powered, prospective randomized trials remains warranted.

## Figures and Tables

**Table 1:** Baseline characteristics of CSP vs. BIV upgrade patients.

Clinical Variable	CSP (*n* = 26)	BIV (*n* = 37)	*p*
Age (years)	75.9 ± 6.3	75 ± 7.4	0.77
Male, *n* (%)	18.0 (72)	32 (86.5)	0.17
VP burden at baseline	78.4 ± 30.2	88 ± 15.3	0.70
Comorbidities, *n* (%)			
Hypertension	25 (100)	36 (97.3)	0.49
Diabetes mellitus	11 (44)	25 (67.6)	0.49
Ischemic cardiomyopathy	13 (50)	15 (40.5)	0.62
Non-ischemic cardiomyopathy	13 (50)	22 (59.5)	0.62
COPD	6 (24)	2 (5.4)	1.00
Stroke/TIA	4 (16)	2 (5.4)	1.00
CKD *	6 (24)	2 (5.4)	0.05
Paroxysmal AF	4 (16)	5 (13.5)	0.33
Persistent AF	2 (8)	2 (5.4)	1.00
Permanent AF	6 (24)	12 (32.4)	0.60
NYHA class I	4 (15.3)	3 (8.1)	0.43
NYHA class II	8 (30.8)	21 (56.8)	0.07
NYHA class III	12 (46.8)	13 (35.1)	0.43
NYHA class IV	2 (7.7)	0 (0)	0.16
Primer pacing indication, *n* (%)			
AV block	25 (96.15)	28 (75.67)	0.07
SSS	3 (12)	9 (24.3)	0.34
ECG characteristics			
QRS duration (ms)	163.3 ± 27.7	170.3 ± 23.9	0.22
LBBB, *n* (%)	6 (24.0)	3 (8.1)	0.14
RBBB, *n* (%)	3 (12.0)	2 (5.4)	0.29
NIVCD, *n* (%)	1 (4.0)	1 (2.7)	1.00
Ventricular pacemaker rhythm, *n* (%)	16 (64.0)	32 (86.5)	0.02
Echocardiographic parameters			
LVEF (%)	34.5 ± 8.0	29.7 ± 7.6	0.01
LVEDD (mm)	57.0 ± 9.0	58.5 ± 6.8	0.45
LAD (mm)	57.9 ± 5.9	60.4 ± 7.9	0.16
RAD (mm)	57.3 ± 6.1	59.6 ± 7.0	0.19
TAPSE (mm)	16.5 ± 3.3	18.5 ± 4.3	0.06
PSAP (mmHg)	39.9 ± 13.7	45.1 ± 12.1	0.13
Grade I of TR, *n* (%)	14 (53.8)	17 (45.9)	0.60
Grade II of TR, *n* (%)	11 (42.3)	15 (40.5)	1.00
Grade III of TR, *n* (%)	1 (3.8)	5 (13.5)	0.38
Baseline medication, *n* (%)			
ACEI/ARB/ARNI	24 (96.0)	35 (94.6)	0.50
Beta blockers	25 (100.0)	35 (94.6)	1.00
SGLT2i	18 (72.0)	31 (83.8)	0.12
MRA	10 (40.0)	19 (51.4)	0.39
Loop diuretic	17 (68.0)	23 (62.2)	0.27
APT	10 (40.0)	11 (29.7)	0.58
Anticoagulant	23 (92.0)	25 (67.6)	0.75
Statins	17 (68.0)	29 (78.4)	0.29

Note: Values are given as mean ± SD or *n* (%). Abbreviations: ACEI, angiotensin-converting enzyme inhibitor; AF, atrial fibrillation; ARB, angiotensin II receptor blocker; ARNI, angiotensin receptor–neprilysin inhibitor; APT, antiplatelet therapy; AV, atrioventricular; BIV, Biventricular pacing; CSP, Conduction system pacing; CKD, chronic kidney disease; COPD, chronic obstructive pulmonary disease; ECG, electrocardiogram; LAD, left atrium diameter; LBBB, left bundle branch block; LVEDD, left ventricular end diastolic diameter; LVEF, left ventricular ejection fraction; MRA, mineralocorticoid receptor antagonist; NIVCD, nonspecific interventricular conduction disease; NYHA, New York Heart Association classification for heart failure; PASP, pulmonary arterial systolic pressure; RAD, right atrium diameter; RBBB, right bundle branch block; SSS, sick sinus syndrome; SGLT2i, sodium–glucose co-transporter 2 inhibitor; TAPSE, tricuspid annular plane systolic excursion; TIA, transient ischemic attack; TR, tricuspid regurgitation; VP, ventricular pacing; * Glomerular filtration rate < 60 mL/min/1.73 m^2^.

**Table 2:** Characteristics of upgrade procedure, lead parameters, electrocardiogram and echocardiogram at the time of implant and follow-up in patients undergoing CSP vs. BIV upgrade.

Parameters	CSP (*n* = 26)	BIV (*n* = 37)	*p*
Procedure time (min)	64.8 ± 7.4	68.4 ± 26.8	0.37
Fluoroscopy time (min)	7.1 ± 7.3	8.6 ± 5.1	0.03
Number of leads implanted	1.2 ± 0.4	1.3 ± 0.5	0.69
More than one lead implanted, *n* (%)	6 (23.1)	10 (27)	0.77
New generator implanted, *n* (%)	17 (65.38)	37 (100)	0.004
ICD generator, *n* (%)	6 (23.1)	17 (45.9)	0.11
Baseline LV/CSP threshold (V) @ 0.5 ms	1.2 ± 0.6	1.2 ± 0.8	1.00
Baseline LV/CSP impedance (Ohm)	626.3 ± 235.8	713.1 ± 303.5	0.54
Follow-up LV/CSP threshold (V) @ 0.5 ms	0.9 ± 0.5	1.2 ± 0.7	0.06
Follow-up LV/CSP impedance (Ohm)	521.8 ± 108.0	587.1 ± 185.3	0.40
Baseline QRS (ms)	163.3 ± 27.7	170.3 ± 23.9	0.22
Follow-up QRS (ms)	132.3 ± 14.2	139.2 ± 17.7	0.14
Baseline NYHA class	2.5 ± 0.9	2.3 ± 0.6	0.61
Follow-up NYHA class	2.0 ± 0.5	2.1 ± 0.9	0.89
Baseline LVEDD (mm)	57.0 ± 9.0	58.5 ± 6.8	0.45
Follow-up LVEDD (mm)	53.2 ± 8.6	54.8 ± 7.8	0.49
Baseline LVEF (%)	34.5 ± 8.0	29.7 ± 7.6	0.01
Follow-up LVEF (%)	40.1 ± 8.9	39.3 ± 13.2	0.45

Note: Values are given as mean ± SD or *n* (%). BIV, biventricular pacing; CSP, conduction system pacing; ICD, implantable cardioverter defibrillator; LV, left ventricular lead; LVEF, left ventricular ejection fraction; min, minutes.

**Table 3:** Electrocardiographic characteristics and type of achieved conduction system pacing.

Parameters	CSP (*n* = 26)
V6RWPeak time (ms)	82.5 ± 15.9
Presence of R’ in V1 (v1R’)	23 (88.5%)
His bundle pacing	2 (7.7%)
Left anterior fascicular pacing	2 (7.7%)
Left septal fascicular pacing	9 (34.6%)
Left posterior fascicular pacing	5 (19.2%)
Left ventricular septal pacing	7 (26.9%)

Note: Values are given as mean ± SD or *n* (%). V6RWPeak time; V6 R wave peak time.

**Table 4:** Hazard ratios for hard clinical outcomes after CSP vs. BIV upgrade.

Outcome	HR (CSP vs. BIV)	95% CI	*p*-Value
All-Cause Mortality	1.74	0.35–8.73	0.50
HF Hospitalization	0.42	0.05–3.82	0.44
HF Hospitalization (Adjusted for baseline LVEF)	0.66	0.07–6.43	0.72
Composite (ACM + HFH)	0.87	0.22–3.51	0.85

Note: Cox proportional hazards regression models were used to estimate hazard ratios (HR) and 95% confidence intervals (CI) for each outcome. All models were unadjusted, except for HFH, which was adjusted for LVEF. The composite endpoint was defined as the first occurrence of either all-cause mortality (ACM) or heart failure (HF) hospitalization.

**Table 5:** Quality-of-life measures after upgrade to conduction system pacing (CSP) or biventricular pacing (BIV).

Quality of Life	CSP	BIV	*p*
Self-reported Health on a scale 0–100	46.5 ± 34.9	41.6 ± 31.9	0.46
Any level of issue with mobility, *n* (%)	11 (42.3)	20 (54.1)	1.00
Any level of issue with self care, *n* (%)	3 (11.5)	8 (21.6)	1.00
Any level of issue with usual activities, *n* (%)	6 (23.1)	8(21.6)	0.95
Any level of pain/discomfort, *n* (%)	5 (19.2)	15 (40.5)	0.85
Any level of anxiety or depression, *n* (%)	3 (11.5)	8.0 (21.6)	1.00

Note: Values are given as mean ± SD or *n* (%).

## Data Availability

Data are available upon request from the corresponding author.

## Abbreviations

The following abbreviations are used in this manuscript:

ACEI	angiotensin-converting enzyme inhibitor
ACM	all-cause mortality
AF	atrial fibrillation
ANCOVA	analysis of covariance
APT	antiplatelet therapy
ARB	angiotensin II receptor blocker
ARNI	angiotensin receptor–neprilysin inhibitor
AV	atrioventricular
BIV	biventricular pacing (cardiac resynchronization therapy)
CIED	cardiac implantable electronic device
CKD	chronic kidney disease
CSP	conduction system pacing
HBP	His-bundle pacing
HFH	heart failure hospitalization
ICD	implantable cardioverter defibrillator
LAD	left atrial diameter
LBBB	left bundle branch block
LBBAP	left bundle branch area pacing
LV	left ventricular
LVEDD	left ventricular end-diastolic diameter
LVEF	left ventricular ejection fraction
MRA	mineralocorticoid receptor antagonist
NIVCD	nonspecific interventricular conduction disease
NYHA	New York Heart Association
PASP	pulmonary arterial systolic pressure
PICM	pacemaker-induced cardiomyopathy
QoL	quality of life
RAD	right atrial diameter
RBBB	right bundle branch block
RV	right ventricular
SDL	stylet-driven leads
SGLT2i	sodium–glucose co-transporter 2 inhibitor
SSS	sick sinus syndrome
TAPSE	tricuspid annular plane systolic excursion
TIA	transient ischemic attack
TR	tricuspid regurgitation
V6RWPeak time	V6 R-wave peak time
VP	ventricular pacing
ΔLVEF	change in left ventricular ejection fraction
